# Ectopic 3rd Molar Tooth in the Maxillary Antrum

**DOI:** 10.1155/2014/620741

**Published:** 2014-07-15

**Authors:** Seidu A. Bello, Ifeoluwa O. Oketade, Otasowie D. Osunde

**Affiliations:** ^1^QH Specialist Dental Clinics and Research Centre, Gwarimpa, Abuja, Nigeria; ^2^Maxillofacial Unit, Department of Dental Surgery, University of Calabar Teaching Hospital, Calabar, Nigeria

## Abstract

Location of ectopic tooth in a nondentate area like the maxillary antrum is rare. A 17-year-old boy, with one year history of recurrent right facial swelling and radiographic finding of a maxillary third molar tooth located at the posterior wall of the maxillary antrum, is presented. Under endotracheal intubation, the tooth was extracted through a Caldwell-Luc antrostomy approach and patient had an uneventful recovery and has been symptom free for eight months. Ectopic tooth in the maxillary antrum is rare and is commonest with maxillary third molar. It may be symptomless but is more commonly associated with inflammatory symptoms. The treatment of choice is surgical excision which is mostly carried out with Caldwell-Luc approach, even though endoscopic approach is being reported.

## 1. Introduction

Maxillary antrum is an uncommon location of an ectopic tooth. Only thirty cases were reported in a medline search between 1980 and 2010 [1]. Few cases have been added to the literature in recent studies [2, 3].

Odontogenesis (tooth development) begins in the sixth week in utero at the time of maxillary and mandibular dental lamina formation [1]. It undergoes complex multistep interaction between the oral epithelium and the underlying mesenchymal tissue resulting in a mature tooth comprising the crown and the root [4, 5]. The ectodermal structure changes to form the enamel while the dentine, pulp, cementum, and surrounding bone are formed by the mesenchyme [5].

Tooth eruption process is, in most cases, a passive one but an abnormal tissue interaction during odontogenesis may result in ectopic tooth development and eruption [2]. The displacement of the tooth may also be due to pressure caused by a cystic enlargement. Another etiology may include developmental disorders such as cleft palate, trauma causing displacement of the teeth, maxillary infection, crowding, genetic factors, and high bone density [6–8].

Ectopic tooth has been rarely reported and this report is being added to the literature to contribute to the body of knowledge on the topic.

## 2. Case Presentation

**Figure 1:**
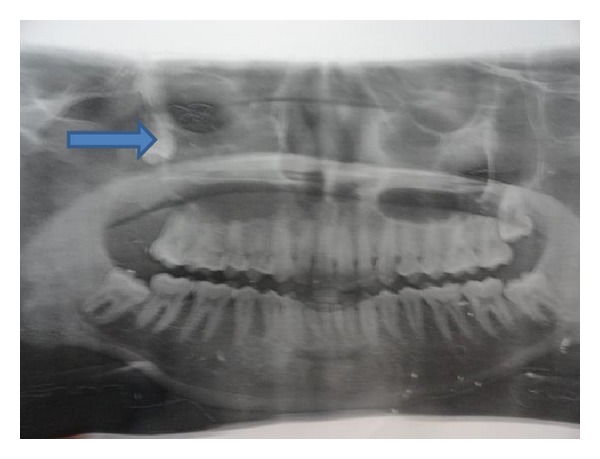
Orthopantomograph showing an ectopic third molar in the right maxillary sinus.

A 17-year-old student was referred to the Oral and Maxillofacial Surgery unit, on account of an accidental radiologic (Orthopantomograph-OPG) discovery of an ectopic tooth in the right maxillary antrum (Figure 1). There was a year history of recurrent right facial pain and swelling.

Examination revealed a right facial swelling, mild pain, and a discharging sinus from the right upper buccal sulcus in relation to tooth number 16. There was full complement of teeth on that arch except tooth number 18. All the teeth were firm and noncarious. The patient had experienced 3 episodes of infection in the past one year. The patient was not HIV positive and there was no history of diabetic mellitus or sickle cell disease. Further systemic examination revealed no abnormality.

**Figure 2:**
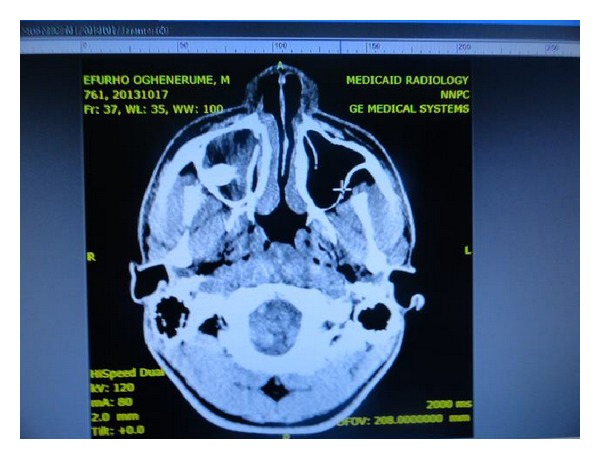
CT scan showing location of an ectopic tooth in the posterior wall of right maxillary antrum.

Further radiological investigation included CT scan as well as Occipitomental and True Lateral of the Skull. Investigation revealed that a molar tooth was located at the posterior wall of the antrum surrounded by radiopacity and no sign of a cyst (Figure 2).

**Figure 3:**
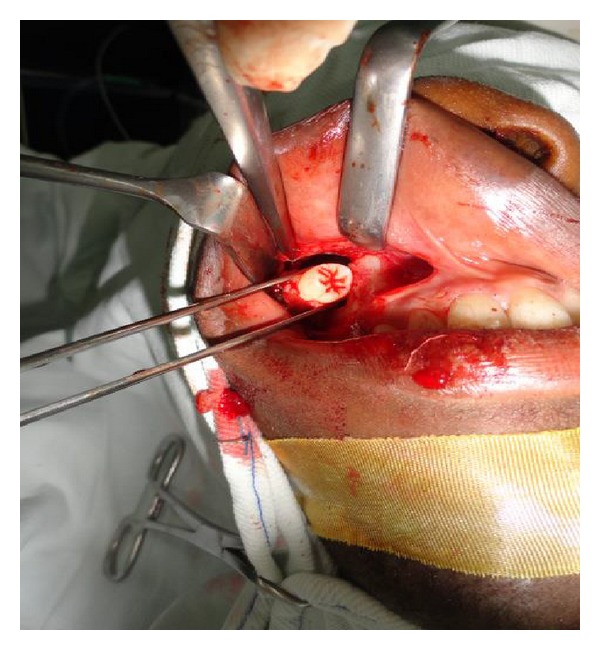
Ectopic third molar tooth, extracted and being delivered through Caldwell-Luc antrostomy.

Through Caldwell-Luc antrostomy approach under endotracheal intubation, the tooth was extracted, pus was evacuated, antrum was properly irrigated, and wound was closed with a resorbable suture with an antral pack in place, which was removed 3 days later (Figure 3). The healing was uneventful and patient has been symptom free for eight months.

## 3. Discussion

Ectopic eruption of a tooth within the dentate region is often seen in clinical practice, which is more common in mandible but such a condition in a nondentate area like maxillary sinus is very rare [9, 10]. Ectopic and supernumerary teeth have also been described in nondental and nonoral sites such as the mandibular condyle, coronoid process, orbit, palate, nasal cavity, nasal septum, and the chin [11]. Beriat et al. [1], in their review of ectopic teeth in maxillary sinus, reported 18 molars, of which 17 were third molars, 5 canine, 3 supernumerary, 1 odontoma, 1 tooth-like structure, and only 1 premolar. The case under consideration is a third molar. The 3rd molar is the last tooth to erupt in the maxilla hence more likely to be affected by displacement while competing for space and this could be responsible for the high incidence of ectopic 3rd molar in the maxilla.

Maxillary teeth in maxillary sinus may be discovered accidentally, precipitate sinusitis, or sometimes result in ophthalmic symptoms [10]. Somayaji et al. [3] in the report of a tooth in the maxillary antrum reported nasal obstruction, discharge, and minimal fullness in right nasolabial area, which is similar to the present case. Inflammatory reaction to the tooth in the antrum, which is foreign to the location, appears to be the commonest presenting symptom. About half of the thirty cases of teeth in the maxillary antrum in Beriat's description also presented with inflammatory symptoms. Foreign bodies (rhinoliths), infections like syphilis, tuberculosis, or fungal infections with calcification, benign lesions such as hemangioma, osteoma, enchondroma, calcified polyp, and dermoid cysts or tumors, and malignant lesions such as chondrosarcoma and osteosarcoma must be considered in the differential diagnosis of ectopic teeth [1].

Water's view, OPG, and lateral cephalogram are simple and inexpensive projections for radiographic evaluation of an ectopic tooth in the maxillary sinus. Though expensive, CT and MRI certainly have an edge over conventional radiographs [12]. In addition to screening investigation with OPG in the case under consideration, CT scan (Figure 2) demonstrated clearly the location of the ectopic tooth at the posterior wall of the antrum, which was important for planning of the extraction. CT scan provides superior bony detail, helps in determination of the size and extent of the lesion, and is useful to distinguish a maxillary lesion of antral origin from an extraantral lesion [13]. Bonder et al. [14] studied 12 patients with teeth in the maxillary sinus by plain film radiography (PFR) and by CT with a dental software program. He found that CT was superior to PFR to determine proximity of the tooth to the sinus wall or its ankylosis, proper surgical planning (crestal incision or Caldwell-Luc approach), as well as prediction of prognosis or complications.

The treatment of an ectopic tooth in the maxillary sinus is removal, as it may lead to cyst formation if left untreated [4]. The traditional approach is Caldwell-Luc procedure, which allows a direct view into the maxillary sinus [12]. This approach was employed in this case which allowed direct access to the tooth as well as meticulous antral irrigation. In another study of 30 cases, the most common approach was Caldwell-Luc procedure (18 cases), five patients were treated with endoscopic sinus surgery, 3 with marsupialization, 2 with crestal incision, and enucleation method was used for only one patient [1]. Somayaji et al. [3] extracted a tooth with endoscopic procedure through sublabial approach and concluded that it enabled better exposure, good illumination, and magnification resulting in less morbidity, meticulous surgery, and faster postoperative recovery. However no direct comparative study was performed in the presentation.

In conclusion, ectopic tooth in the maxillary antrum is rare and is commonest with maxillary third molar. It may be symptomless but is more commonly associated with inflammatory symptoms. The treatment of choice is surgical excision which is mostly carried out with Caldwell-Luc approach, even though endoscopic approach is being reported.
